# Platelet membrane-camouflaged matrine biomimetic nanoparticles for efficient acute lung injury therapy

**DOI:** 10.1016/j.isci.2026.114683

**Published:** 2026-01-13

**Authors:** Yinqiang Fan, Haiyuan Zhang, Xingxing Chai, Jiahao Liu, Yinlian Yao, Xin Shen, Ru Li, Ning Wei, Meiting Yang, Lixia Li, Xiaolei Zhao, Yue Zhao, Hua Jin

**Affiliations:** 1 The First Dongguan Affiliated Hospital, Guangdong Medical University, Dongguan 523808, China; 2 Feicheng People Hospital, Taian 271600, China; 3 Guangdong Provincial Key Laboratory of Research and Development of Natural Drugs, School of Pharmacology, Laboratory Animal Center, Guangdong Medical University, Dongguan 523808, China; 4 The Key Laboratory of Medical Immunology and Molecular Diagnostics of Guangdong Province, Dongguan Key Laboratory of Medical Bioactive Molecular Developmental and Translational Research, Guangdong Medical University, Dongguan 523808, China

**Keywords:** Therapeutics, drug delivery system, pharmacology, nanoparticles, biological sciences, materials science, Biomaterials

## Abstract

Sepsis frequently progresses to acute lung injury (ALI) and acute respiratory distress syndrome (ARDS), which are associated with high mortality. Current treatments face limitations due to resistance and toxicity. Matrine, a component of traditional Chinese medicine, exhibits anti-inflammatory properties but suffers from poor selectivity and potential toxicity. To address these challenges, this study developed platelet membrane-coated matrine nanoparticles (PM@Mat-NPs) for targeted ALI treatment. The PM@Mat-NPs, characterized by a particle size of ∼500 nm, zeta potential of −58 mV, and encapsulation efficiency of 82.1 ± 1.5%, effectively inhibited LPS-induced endothelial cell damage *in vitro*. In an ALI mouse model, PM@Mat-NPs selectively accumulated in inflamed lung tissue, significantly reduced pro-inflammatory cytokines (TNF-α, IL-6), and alleviated histological damage. They also suppressed NLRP3 inflammasome activation and apoptosis, outperforming free matrine. Biosafety evaluations confirmed no significant adverse effects. This study introduces a biomimetic nano-platform for the targeted delivery of Chinese herbal components, offering a promising strategy for treating acute lung injury.

## Highlights

• Mat-NPs achieve efficacy enhancement and toxicity in ALI treatment
• The property of platelets enables nanoparticles to target lung inflammation
• Nasal administration is convenient and reduces the systemic toxic side effects
• The abundance and easy accessibility of platelets favor their clinical translation

## Graphical abstract

**Figure:**
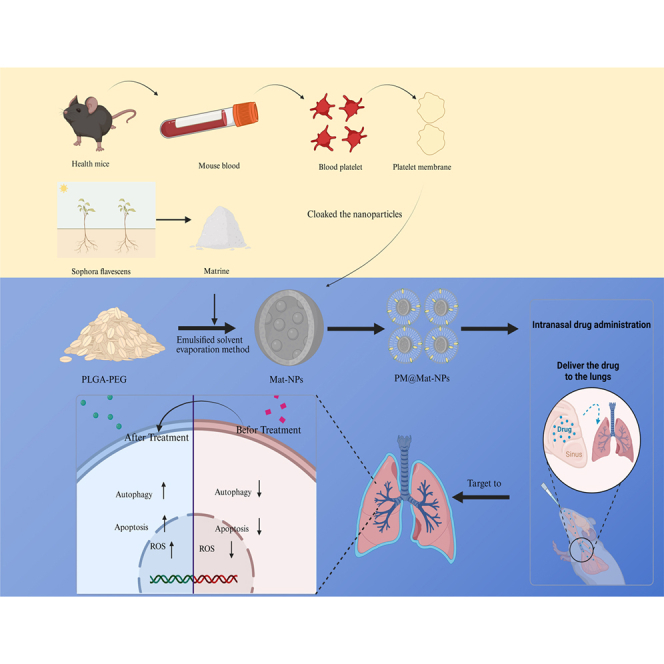

## Introduction

Sepsis, a life - threatening medical condition characterized by organ dysfunction arising from a dysregulated host immune response to infection, imposes a significant financial burden on healthcare systems worldwide.^1^ It is also a leading cause of mortality among patients in the intensive care unit (ICU). Notably, pneumonia is the most prevalent infection in sepsis cases,^2^ often leading to the development of acute lung injury (ALI), and in more severe instances, acute respiratory distress syndrome (ARDS).

Over the past few decades, the clinical options for treating sepsis have been relatively limited, primarily relying on pharmacological interventions and ventilatory support.^3^ The early administration of antibiotics is a cornerstone of sepsis treatment, as it eliminates bacteria and prevents organ damage.^4^ However, misuse or overuse of antibiotics can lead to drug resistance and complicate treatment. Glucocorticoids, another pharmacological intervention, inhibit the NF-κB signaling pathway, thereby reducing pro-inflammatory cytokines such as IL-1 and IL-6 while promoting the production of IL-10, thus exerting potent anti-inflammatory effects. Nevertheless, high-dose “pulse therapy” with glucocorticoids can lead to severe toxicities and organ dysfunction.^5^ Currently, low-tidal-volume ventilation support therapy is the only effective clinical method for reducing mortality rates in sepsis. Yet, despite advancements in invasive diagnostic and treatment methods, ALI and its complications remain prevalent, with mortality rates not significantly decreasing in recent decades.^6^ Therefore, the exploration of more natural and less toxic supplementary and alternative therapeutic agents, as well as the development of biomimetic locally targeted treatment techniques and methods, is of paramount clinical urgency to control medication use and reduce drug resistance and toxic side effects during the treatment of patients with sepsis.

Many traditional Chinese medicine (TCM) monomers are derived from natural sources, such as plants, animals, or minerals. Compared with chemically synthesized drugs, these natural compounds generally exhibit superior safety and tolerability within the human body. A substantial body of research has demonstrated the potential benefits of TCM monomers in mitigating inflammation, modulating immune responses, and enhancing organ function in patients with septic pneumonia.^7^ Matrine, a natural alkaloid extracted from the rhizome of the Sophora flavescens plant, possesses a wide range of pharmacological properties, including anti-allergic, anti-inflammatory, antiviral, and anti-fibrotic activities.^8^ Mat effectively suppresses cytokine storms by targeting molecular interactions and modulating signaling pathways, while simultaneously downregulating pro-inflammatory cytokines such as IL-1β, TNF-α, and MIP-3α. These characteristics render Mat a promising candidate for the prevention and treatment of cytokine release syndrome (CRS) and related disorders.^9^ However, systemic administration (e.g., oral or intravenous) of matrine at therapeutically effective doses often causes off-target toxicities (hepatotoxicity, neurotoxicity) due to its non-specific distribution, which limits clinical translation. Lacking specific recognition of inflamed lung tissues, matrine distributes widely in normal organs and tissues. This non-selectivity reduces its therapeutic concentration at the lesion site and elevates the risk of adverse effects in non-target organs, necessitating high doses that may further exacerbate hepatotoxicity, neurotoxicity, and developmental toxicity, as reported in previous studies.

To address these limitations, nano-delivery systems offer unique advantages. Nanoparticles can encapsulate matrine to improve its stability and control its release, thereby reducing off-target accumulation. More importantly, cell-membrane coated nanoparticles, especially platelet membrane-coated ones, provide a biomimetic solution to enhance targeting. These carriers integrate natural cell membranes with synthetic nanomaterials, thereby synergistically enhancing biocompatibility and mitigating the risk of immune rejection.^10^ The design of these biomimetic nano-drug carriers involves the strategic “coating” of nanoparticles with cell membranes. This innovative approach leverages the inherent biological activity of membrane proteins present in the cell membranes. Specifically, the membrane proteins play a crucial role in facilitating immune evasion, a critical factor in ensuring the successful delivery of therapeutic agents to the target site. Moreover, these proteins contribute to extending the circulation time of the nanoparticles within the body, thereby increasing the likelihood of effective drug delivery. Additionally, the biomimetic property of these carriers enables specific targeting for precise drug delivery to desired tissues or cells.^11^ Compared with gene coating or copper coating,^12^^,^^13^ our platelet membrane-coated PM@Mat-NPs exhibit unique advantages in targeting inflamed lung tissues for acute lung injury treatment.

Platelets, crucial for hemostasis, possess surface proteins that endow unique properties to the nanoparticles they coat. The platelet membrane contains various adhesion molecules, such as integrins and selectins.^14^ These molecules enable the platelet membrane-coated nanoparticles to specifically recognize and bind to inflamed endothelial cells in the lung. Besides, we chose platelet membranes primarily due to their unique advantages over leukocytes or macrophages.^15^ Platelets are extremely abundant (over 100,000/μL blood) and easily obtainable from blood, simplifying large-scale membrane isolation. As anucleate cells, their membrane extraction is simpler without nuclear contamination, unlike nucleated leukocytes/macrophages. Importantly, autologous platelets avoid immune rejection risks associated with allogeneic leukocyte/macrophage membranes. These features make platelet membranes more practical for biomimetic nanoparticle fabrication.

This targeted delivery not only increases the local concentration of matrine in the injured lung but also minimizes its distribution in other organs, thereby reducing systemic toxicity. By combining the anti-inflammatory properties of matrine with the targeting and biocompatibility advantages of platelet membrane-coated nanoparticles, the PM@Mat-NPs system is designed to overcome the key deficiencies of matrine, providing a more effective and safer therapeutic strategy for ALI.

In this study, we aimed to evaluate the therapeutic potential of Mat (Mat) in ALI and sepsis treatment. Initially, we employed network pharmacology to elucidate the mechanisms underlying Mat’s anti-inflammatory effects. Our analysis revealed that Mat functions as a calcium antagonist, mitigating inflammation in ALI by suppressing intracellular calcium levels and inhibiting mitochondrial function in macrophages. To enhance the bioavailability and therapeutic efficacy of Mat, we subsequently developed an intranasal nanoparticle delivery system utilizing platelet membrane-coated nanoparticles (PM@Mat-NPs) for targeted delivery to a murine model of ALI. Comprehensive *in vitro* and *in vivo* experiments were conducted to assess the therapeutic effects of both Mat and PM@Mat-NPs. Our results demonstrated that PM@Mat-NPs selectively accumulated in inflamed lung tissues, effectively scavenged inflammatory cytokines, and reduced cell apoptosis. These findings collectively indicate that the PM@Mat-NP delivery system exhibits excellent biocompatibility and holds significant potential for clinical translation and personalized treatment strategies in ALI.

## Results and discussion

### Analysis of the network pharmacology of Mat

#### Radial network visualization

**Figure 1:**
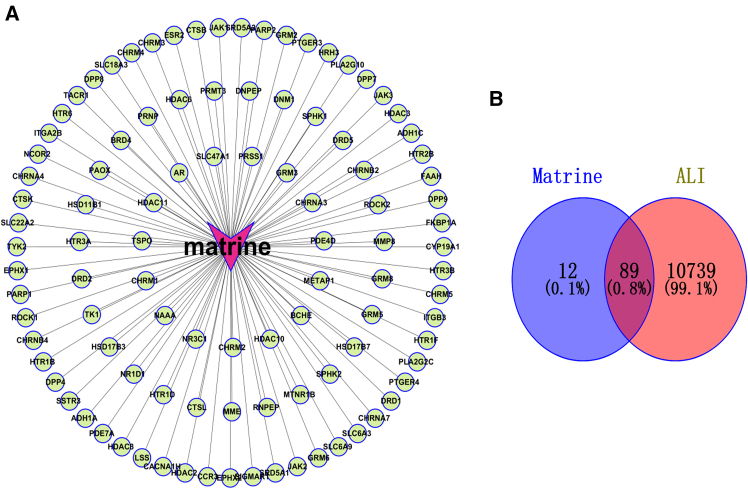
Network pharmacology analysis of Mat and ALI (A) Network diagram depicts the interactions between Mat and its target points. (B) Venn diagram illustrates the shared targets between Mat and ALI.

Figure 1A presents a radial network centered on Mat, highlighting its pivotal role in interacting with a diverse array of biological entities, including targets, genes, and proteins. The connecting lines between Mat and these entities suggest potential direct binding or regulatory effects, underscoring its broad range of action targets in biological processes.

#### Target overlap analysis

Figure 1B employs a Venn diagram to compare the targets associated with Mat and ALI. The intersection reveals 89 shared targets (0.8%), while Mat and ALI have 12 (0.1%) and 10,739 (99.1%) unique targets, respectively. This limited but potentially significant overlap between Mat’s targets and those involved in ALI suggests that Mat may selectively modulate key pathways relevant to ALI pathogenesis.

#### Reorganized radial network

**Figure 2:**
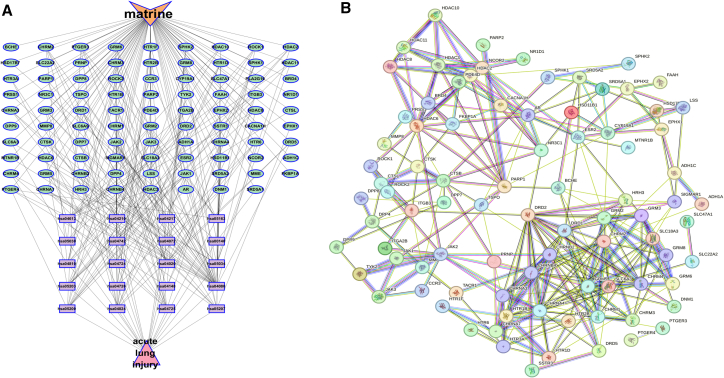
Network analysis of Mat and ALI (A) Mat - shared target genes - pathway - ALI network diagram. The orange node represents Mat, the green nodes represent shared target genes, the purple nodes represent KEGG pathways, and the pink nodes represent ALI. (B) Shared target protein network diagram of Mat and ALI.

Figure 2A reorganizes the radial network from Figure 1A, maintaining Mat’s central position while emphasizing its interactions with specific targets. This layout clarifies the relationship network, potentially highlighting Mat’s role in specific biological pathways and elucidating its multifaceted mechanisms of action.

#### Complex interaction network

Figure 2B depicts a complex network with nodes representing various biological entities and colored lines indicating diverse interactions. This intricate structure reflects Mat’s involvement in multiple biological processes and signaling pathways, suggesting a multifaceted mechanism of action. The interconnections among these entities highlight the complexity of Mat’s biological impact.

#### Hierarchical network structure

**Figure 3:**
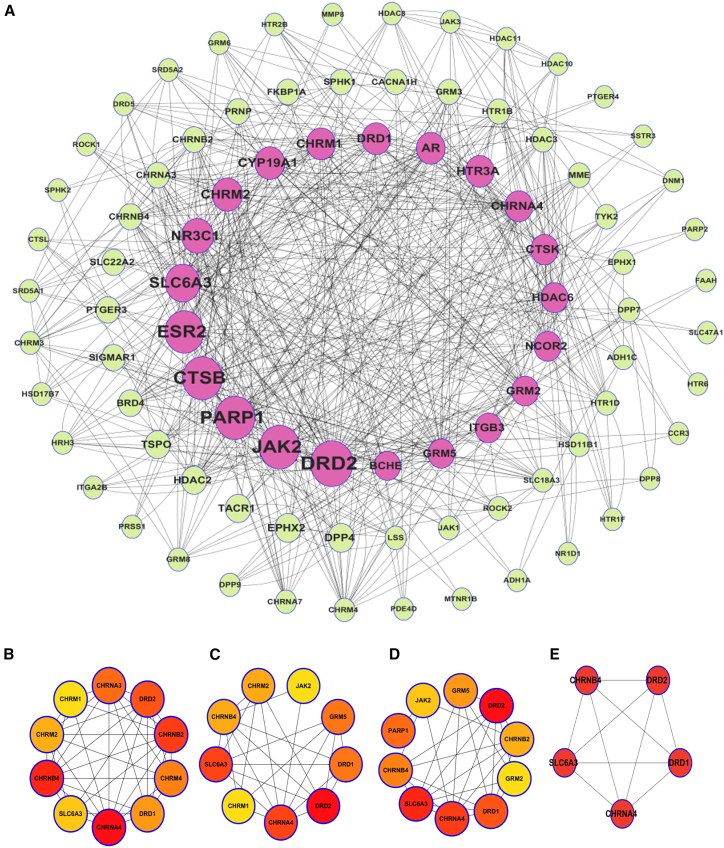
Protein-protein interaction (PPI) network and topological analysis of key targets (A) Protein-protein interaction (PPI) network. (B–D) Topological analysis of key targets in the PPI network: (B) PPI network diagram obtained using the MCC algorithm. (C) PPI network diagram obtained using the Degree algorithm. (D) PPI network diagram obtained using the Closeness algorithm. (E) PPI network diagram of key targets identified by the Betweenness centrality algorithm.

Figure 3A presents a circular network, with densely connected core nodes linked to peripheral nodes. This hierarchical arrangement illustrates how core targets under Mat’s influence regulate peripheral molecules, revealing a potential mode of action. The dense interconnections among core nodes suggest that Mat may exert its effects through a coordinated regulation of key biological pathways.

#### Modular network organization

Figures 3B–3D show multiple smaller circular networks, each representing distinct biological processes or functional modules. These structures suggest that Mat exerts its effects by regulating various functional modules, highlighting its diverse biological impacts. The modular organization underscores Mat’s potential to modulate multiple pathways simultaneously, which is critical for its therapeutic efficacy in complex diseases such as ALI.

#### Key module identification

Figure 3E simplifies the network to a key module with red nodes, likely representing crucial targets or molecules. Their interconnections may explain a core mechanism or functional unit underlying Mat’s effects. This key module highlights the essential pathways and targets that are most relevant to Mat’s therapeutic potential.

#### Functional enrichment analysis

**Figure 4:**
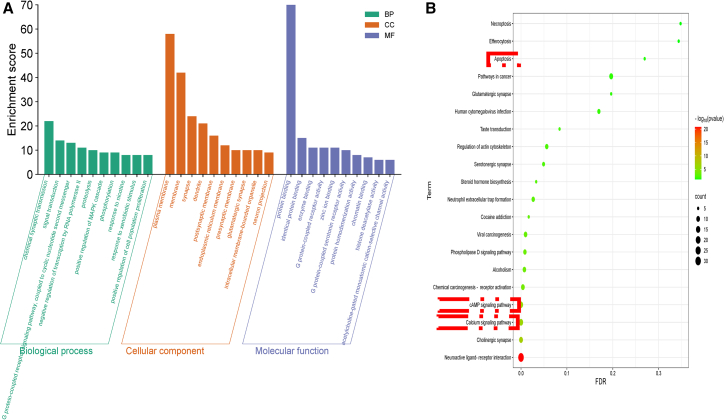
Analysis of signaling pathways associated with Mat (A) Gene Ontology (GO) functional enrichment analysis. This panel illustrates the biological processes, cellular components, and molecular functions affected by Mat. (B) KEGG pathway enrichment bubble chart. This panel displays the significant pathways influenced by Mat, with the bubble size representing the number of associated genes and the color indicating the level of significance.

Bioinformatics analysis of the 89 overlapping targets between Mat and ALI revealed key biological pathways and functional annotations. Gene Ontology (GO) enrichment highlighted synaptic transmission, signal transduction, and G-protein coupled receptor signaling as predominant biological processes (Figure 4A). Cellular components were enriched in plasma membrane and synaptic regions, while molecular functions centered on protein and enzyme binding.

#### Pathway enrichment analysis

KEGG pathway analysis further identified 28 pathways, with neuroactive ligand-receptor interactions, calcium signaling, cAMP signaling, and cholinergic synapse pathways ranking highest (Figure 4B). Notably, pathways in cancer and chemical carcinogenesis were also implicated, suggesting potential cross-talk between Mat’s mechanisms and broader cellular processes.

These analyses show that Mat has a wide interaction network and is a key regulator of many biological processes. The limited but significant overlap with ALI targets suggests potential therapeutic relevance. The hierarchical and modular network structures (Figure 4B) further reveal Mat’s complex mechanism, involving core targets that regulate peripheral molecules and diverse functional modules. The strong enrichment of synaptic and neuroactive ligand-receptor pathways indicates that Mat may modulate neuroimmune crosstalk or neurotransmitter-mediated inflammation, both of which are related to ALI pathophysiology. The prominence of calcium and cAMP signaling pathways further supports Mat’s role in regulating intracellular signaling cascades that are critical for inflammatory responses, endothelial barrier integrity, and oxidative stress mitigation, which are hallmarks of ALI progression. The unexpected association with cancer-related pathways (e.g., chemical carcinogenesis) raises interesting questions. These pathways may reflect off-target effects or indirect regulatory networks, but they could also suggest broader roles for Mat in cellular proliferation or apoptosis regulation, which may indirectly influence ALI resolution. The convergence of multiple signaling axes (e.g., calcium, cAMP, and cholinergic signaling) highlights Mat’s multi-target action, consistent with its complex interaction network. This polypharmacological profile may explain its therapeutic potential in ALI, a multifactorial disease that requires simultaneous modulation of inflammation, vascular permeability, and oxidative damage. Future studies should prioritize functional validation of these pathways, especially the calcium-cAMP crosstalk, to clarify their specific contributions to Mat’s efficacy in ALI models. This analysis positions Mat as a multi-faceted agent with dual regulatory roles in neuroimmune signaling and intracellular second-messenger systems, providing a mechanistic basis for its therapeutic application in ALI.

### Characteristics of Mat-loaded nanoparticles

**Figure 5:**
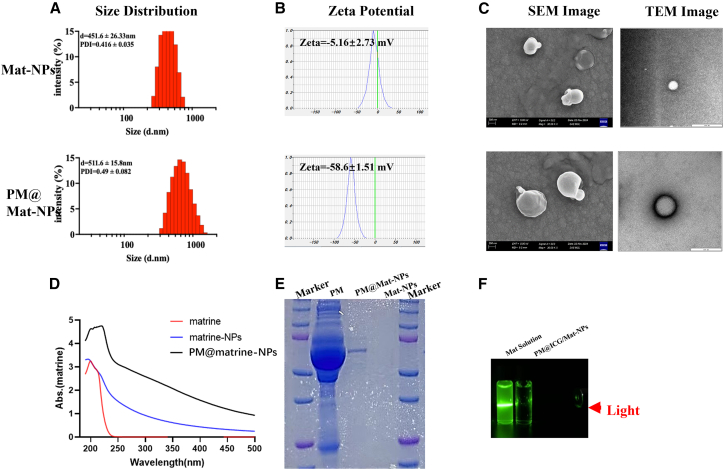
Physicochemical characterization of Mat nanoparticles (Mat-NPs) and PM@Mat-NPs (A) Size distribution analysis. This panel shows the particle size range and uniformity of the synthesized Mat-NPs and PM@Mat-NPs, indicating their size distribution profiles. (B) Surface zeta potential measurements, presenting the zeta potential values of Mat-NPs and PM@Mat-NPs, assessing their surface charge characteristics and stability in suspension. (C) Scanning electron microscopy (SEM) Images, providing visual confirmation of the morphology and surface structure of Mat-NPs and PM@Mat-NPs, revealing their physical appearance at the nanoscale. (D) UV-Vis spectroscopy, which demonstrates the optical properties and absorbance characteristics of Mat-NPs and PM@Mat-NPs, highlighting their specific absorption profiles. (E) The protein electrophoresis substrate gel detection showed that Mat-NPs had no protein expression. The protein expression of PM@Mat-NPs was highly similar to that of the platelet membrane group, indicating that the platelet membrane proteins had been successfully encapsulated onto the nanoparticles. (F) Tyndall effect, illustrating the colloidal stability of the synthesized nanoparticles in suspension, evidenced by the visible scattering of light.

The physicochemical properties of Mat-loaded nanoparticles (Mat-NPs) and platelet membrane-coated biomimetic nanoparticles (PM@Mat-NPs) were thoroughly investigated. Particle size analysis indicated that Mat-NPs exhibited an average diameter of 450 nm, whereas PM@Mat-NPs demonstrated a slightly larger size of approximately 510 nm (Figure 5A). This increase in size is indicative of the successful encapsulation of Mat-NPs by the platelet membrane, thereby enhancing their biocompatibility and targeting capabilities. Zeta potential measurements revealed that Mat-NPs possessed a neutral surface charge of −5 mV, while PM@Mat-NPs exhibited a significantly more negative charge of −58 mV (Figure 5B), confirming the effective adsorption of platelet membrane proteins onto the nanoparticle surface. Scanning electron microscopy (SEM) observations revealed that Mat-NPs exhibited a uniform spherical morphology, whereas PM@Mat-NPs displayed larger and more complex structures due to the additional platelet membrane layer (Figure 5C). UV-Vis spectroscopy confirmed the presence of Mat in both types of nanoparticles (Figure 5D), thereby ensuring their therapeutic efficacy. The protein electrophoresis substrate gel detection showed that Mat-NPs had no protein expression. The protein expression of PM@Mat-NPs was highly similar to that of the platelet membrane group, indicating that the platelet membrane proteins had been successfully encapsulated onto the nanoparticles (Figure 5E). The Tyndall effect demonstrated that PM@Mat-NPs exhibited enhanced colloidal stability and bioavailability compared to Mat solution (Figure 5F). These findings highlight the successful development of a biomimetic nanoparticle system with enhanced targeting and therapeutic properties.

### Impact of Mat on the biophysical and biochemical properties of endothelial cells

#### Regulation of intracellular calcium influx

**Figure 6:**
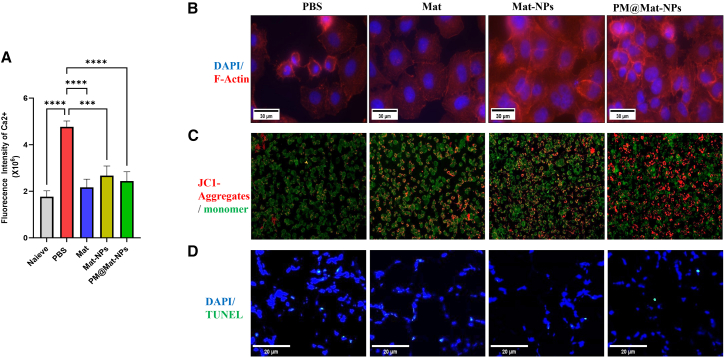
Effects of Mat on biophysical and biochemical features of lipopolysaccharide (LPS)-induced inflammatory endothelial cells (A) Intracellular calcium ion (Ca^2+^) levels. All Mat formulations significantly inhibit Ca^2+^ influx induced by LPS, indicating their role in regulating the calcium signaling pathway and attenuating initial inflammatory signals. (B) Cytoskeletal integrity. Rhodamine-labeled phalloidin staining of the endothelial cell (EC) cytoskeleton reveals that Mat, particularly PM@Mat-NPs, restores F-actin disrupted by LPS, thereby protecting cytoskeletal structure and cellular functions. (C) Mitochondrial function. JC-1 staining for mitochondrial membrane potential demonstrates that Mat restores mitochondrial function and energy metabolism, thereby maintaining cellular energy homeostasis under inflammatory conditions. (D) Apoptosis assessment. TUNEL staining for endothelial cell apoptosis rate shows that different Mat formulations alleviate LPS-induced cell death, inhibiting inflammation-induced apoptosis and protecting endothelial cell survival. Statistical analyses were performed using the two-tailed unpaired Student’s *t* test. ∗∗∗*p* < 0.001, ∗∗∗∗*p* < 0.0001 (*n* = 4).

In the context of inflammatory responses, endothelial cells (ECs) play a pivotal role, particularly in their reaction to lipopolysaccharide (LPS) stimulation. LPS, a potent inflammatory inducer, triggers a cascade of intracellular events, including a significant influx of calcium ions (Ca^2+^), which is a hallmark of early inflammatory activation.^16^ In this study, we investigated the effect of Mat on this Ca^2+^ influx. Our results demonstrated that Mat, especially in the form of platelet membrane-coated nanoparticles (PM@Mat-NPs), effectively inhibited the LPS-induced Ca^2+^ influx (Figure 6A). These finding highlights Mat’s capacity to modulate cellular signaling pathways, thereby attenuating the inflammatory response.

#### Restoration of cytoskeletal integrity

The maintenance of cellular structural integrity is crucial for endothelial cell function.^17^ We employed Rhodamine-labeled phalloidin staining to examine the reorganization of the EC cytoskeleton, specifically focusing on F-actin filaments (Figure 6B). LPS exposure led to the disruption of F-actin filaments, compromising cellular integrity. In contrast, treatment with Mat, particularly PM@Mat-NPs, significantly restored F-actin levels, indicating the preservation of cytoskeletal stability and cellular function.

#### Mitochondrial function and membrane potential

Mitochondrial function is a key determinant of cellular homeostasis and energy metabolism.^18^ We assessed mitochondrial membrane potential using JC-1 staining, a crucial indicator of mitochondrial health. LPS-induced mitochondrial dysfunction was evident through depolarization of the mitochondrial membrane potential. However, Mat treatment effectively reversed this depolarization, restoring mitochondrial membrane potential (Figure 6C). This finding underscores Mat’s role in maintaining mitochondrial integrity and ensuring normal cellular energy homeostasis.

#### Cell viability and apoptosis

Cell viability and apoptosis are critical endpoints in evaluating the impact of LPS and Mat on endothelial cells.^19^ We used TUNEL staining to quantify apoptosis rates in ECs. Results revealed that LPS significantly increased cell death, while various formulations of Mat, including PM@Mat-NPs, effectively alleviated LPS-induced apoptosis (Figure 6D). This demonstrates Mat’s protective effects against inflammatory cell death and its ability to preserve endothelial cell viability.

Mat emerges as a highly promising therapeutic agent for inflammatory-related diseases. Its multifaceted actions, including the regulation of inflammatory responses, protection of cellular functions, and enhancement of cellular survival, highlight its potential as a biomimetic therapeutic strategy. These findings collectively underscore Mat’s significance in modulating key biophysical and biochemical properties of endothelial cells, thereby offering a robust foundation for further exploration in the field of inflammatory disease research.

### *In vivo* targeting and anti-inflammation of platelet membrane-coated matrine nanoparticles

#### Establishment of the acute lung injury model and treatment administration

**Figure 7:**
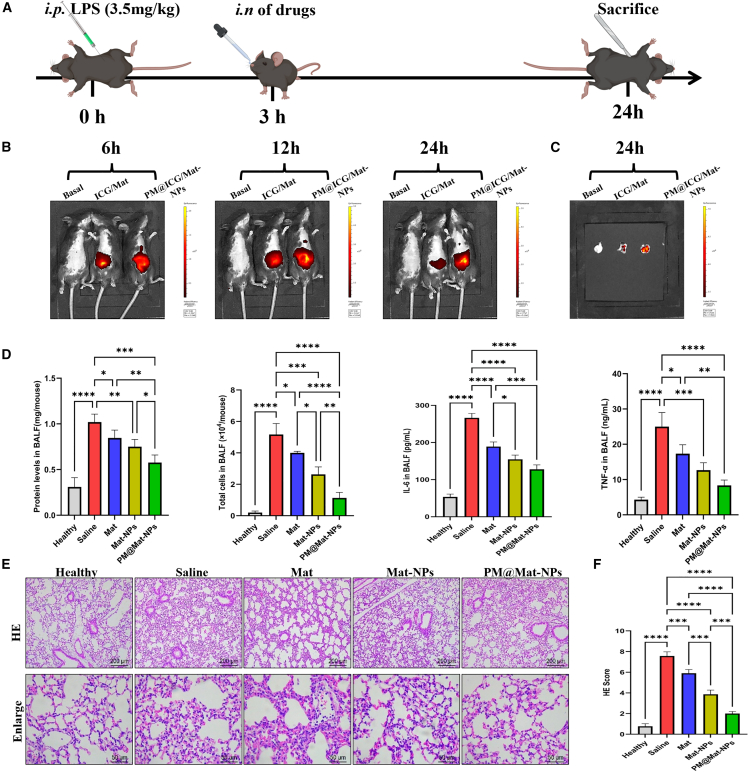
Biodistribution and anti-inflammatory effects of PM-coated biomimetic nanoparticles in ALI mice (A) Experimental protocol timeline: Mice were intraperitoneally (*i.p.*) injected with LPS (3.5 mg/kg body weight) to induce the ALI model. After a 3-h interval, mice received intranasal (i.n.) administration of various treatments. Animals were euthanized for tests at different time periods. (B–C) Biodistribution imaging: Representative images obtained via *in vivo* imaging system (IVIS) show the biodistribution in whole bodies and specifically in lung tissues of ALI mice at 6, 12, and 24 h following *i.n.* of either ICG/Mat or PM@ICG/Mat-NPs. (D) Inflammation levels in bronchoalveolar lavage fluid (BALF): quantitative analysis of protein levels, total cell counts, and pro-inflammatory cytokines -- IL-6 and TNF-α in BALF following treatment. (E–F) Histological examination: Representative micrographs of lung tissue cross-sections taken 24 h post-LPS challenge, stained with H&E, and H&E scores of the sections. Statistical analyses were performed using the two-tailed unpaired Student’s *t* test. ∗*p* < 0.05, ∗∗*p* < 0.01, ∗∗∗*p* < 0.001, ∗∗∗∗*p* < 0.0001 (*n* = 4).

To investigate the therapeutic potential of Mat formulations in ALI, we established a reliable ALI mouse model using intraperitoneal (*i.p.*) injection of lipopolysaccharide (LPS) at a dosage of 3.5 mg/kg body weight. This LPS dosage has been validated to induce a reproducible and clinically relevant inflammatory response in the lungs. After allowing 3 h for the initial inflammatory cascade to develop, mice received intranasal administration of various therapeutic agents, including free Mat, Mat-NPs, and PM@Mat-NPs. This administration route was chosen to directly target the respiratory tract, enhancing the clinical relevance of our findings. Mice were euthanized 24 h post-LPS exposure to capture the peak inflammatory response and assess treatment outcomes (Figure 7A).

#### Biodistribution of platelet membrane-coated matrine nanoparticles

To evaluate the biodistribution of the administered compounds, we utilized the *In Vivo* Imaging System (IVIS), a cutting-edge technology that enables real-time, non-invasive monitoring of nanoparticle distribution within living organisms. IVIS data revealed that intranasally, with PM@Mat-NPs accumulated significantly more in lung tissues compared to free Mat (Figures 7B and 7C). This enhanced accumulation is likely due to the surface modification of nanoparticles with platelet membranes, which facilitates their interaction with inflamed lung tissue. This finding highlights the potential of biomimetic nanoparticles as a targeted delivery system for anti-inflammatory agents in ALI.

#### Anti-inflammatory efficacy assessed by bronchoalveolar lavage fluid analysis

Quantitative analysis of bronchoalveolar lavage fluid (BALF) was conducted to evaluate key indicators of the inflammatory response, including protein levels, total cell counts, and concentrations of pro-inflammatory cytokines such as IL-6 and TNF-α. Elevated protein levels and cell counts in BALF (Figures 7D and 7E) indicate increased vascular permeability and immune cell recruitment, which are characteristic features of ALI. Treatment with Mat or Mat-NPs notably reduced these permeabilities. Furthermore, Figures 7F and 7G demonstrated that Mat treatment significantly decreased the levels of these inflammatory markers (IL-6 and TNF-α), confirming its anti-inflammatory efficacy. Notably, PM@Mat-NPs exhibited the most pronounced anti-inflammatory effects across all assays, likely due to their enhanced biodistribution and targeting capabilities.

#### Histological assessment of lung tissue

Histological examination of lung tissue sections stained with Hematoxylin and Eosin (H&E) was conducted to further evaluate the extent of lung injury and inflammatory cell infiltration. As indicated by the staining and scores of H&E in Figures 7H and 7I, treatment with Mat, and particularly with PM@Mat-NPs, substantially mitigated lung damage and significantly suppressed the degree of inflammatory cell infiltration. These findings corroborate the quantitative data from BALF analysis, underscoring the potent anti-inflammatory efficacy of the treatments, with PM@Mat-NPs demonstrating superior histopathological outcomes likely due to their enhanced biodistribution and targeted delivery capacity.

#### Platelet membrane-coated matrine nanoparticles suppress NLRP3 activation and apoptosis in acute lung injury lungs

**Figure 8:**
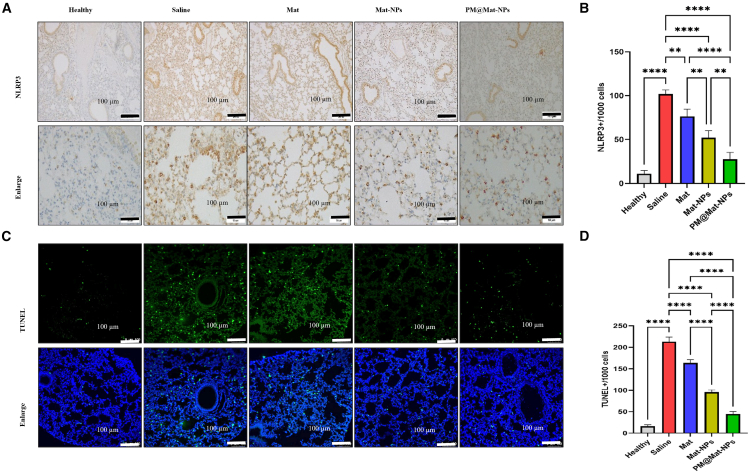
Immunohistochemical analysis of NLRP3 inflammasomes and apoptosis in lung sections of ALI Mice (A–D) Representative images and semi-quantitative evaluations of NLRP3 inflammasome expression (A–B) and apoptotic cells using the TUNEL assay (C–D) in lung sections from mice with ALI. Staining cell counts were quantified to assess NLRP3 levels and apoptosis. Quantitative data are presented as mean ± SEM. Statistical analyses were performed using the two-tailed unpaired Student’s *t* test. ∗*p* < 0.05, ∗∗*p* < 0.01, ∗∗∗*p* < 0.001, ∗∗∗∗*p* < 0.0001 (*n* = 4).

To explore the pathological mechanisms underlying ALI and the therapeutic potential of Mat, we examined the activation of the NLRP3 inflammasome and apoptosis in lung tissues. Immunohistochemical analysis and semi-quantitative evaluation revealed significant activation of the NLRP3 inflammasome in the lung tissues of ALI mice treated with PBS (Figures 8A and 8B). This activation drives the inflammatory cascade in ALI, leading to the release of pro-inflammatory cytokines and exacerbating tissue damage.^20^ In contrast, treatment with Mat, especially PM@Mat-NPs, significantly reduced NLRP3 inflammasome activation, indicating its capacity to modulate this key inflammatory pathway.

TUNEL staining, a gold-standard method for detecting apoptotic cells, revealed a substantial number of apoptotic cells in the lung tissues of ALI mice treated with PBS (Figures 8C and 8D). This indicates severe tissue damage and disruption of cellular homeostasis in ALI. However, treatment with Mat, particularly PM@Mat-NPs, significantly decreased the number of apoptotic cells, suggesting its protective effects against apoptosis and preservation of tissue integrity.

#### Biosafety of Platelet membrane-coated matrine nanoparticles

**Figure 9:**
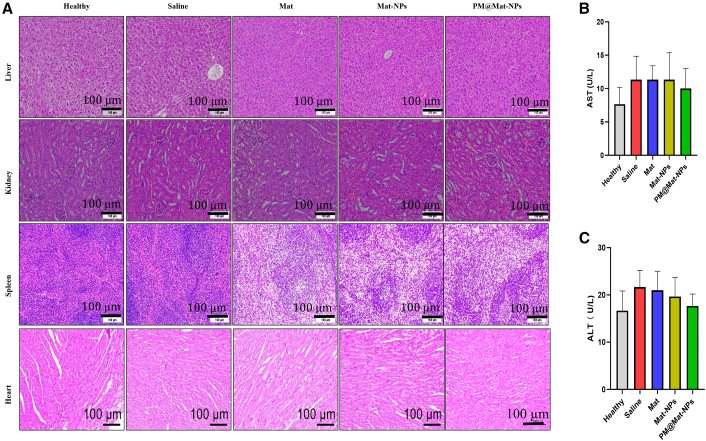
*In vivo* biosafety assessment of various Mat formulations (A) Representative photographs of major organs. ALI mice were administered different Mat formulations intranasally once daily for 7 consecutive days at a dosage of 10 mg/kg body weight. Photomicrographs of major organs provide a visual assessment of potential morphological changes or lesions. Scale bars = 100 μm. (B–C) Biochemical analysis of liver toxicity. Levels of AST and ALT were quantified in the serum of mice from each treatment group to evaluate potential hepatotoxicity.

Ensuring the safety of nanoparticle-based drug delivery systems is crucial for their successful clinical application.^21^ We conducted a comprehensive evaluation of the biosafety of Mat and its nanoparticle formulations in ALI mice. Histological examinations of vital organs, including the liver, kidneys, spleen, and heart, revealed no significant toxicological features in any treated groups (Figure 9A). Additionally, biochemical analysis of liver-related markers, including ALT and AST in the serum (Figures 9B and 9C), indicated no significant hepatotoxicity caused by Mat or its nanoparticle formulations. These findings provide critical safety evidence for further research and potential application of Mat and Mat-NPs in the context of ALI.

In summary, the *in vivo* results unequivocally demonstrate that Mat and its nanoparticle formulations, particularly PM@Mat-NPs, exhibit substantial anti-inflammatory efficacy in an LPS-induced ALI mouse model. The enhanced targeting capability of PM@Mat-NPs, as evidenced by their preferential accumulation in lung tissues, highlights the promise of biomimetic nanoparticles as a targeted delivery platform for anti-inflammatory agents. The marked reduction in inflammatory markers in BALF, preservation of lung tissue integrity, and modulation of NLRP3 inflammasome activation and apoptosis collectively underscore the therapeutic potential of Mat and its nanoparticle formulations. Furthermore, a comprehensive biosafety evaluation confirms the safety profile of PM@Mat-NPs, thereby paving the way for their potential clinical application.

### Conclusions

This study offers a comprehensive exploration of the therapeutic potential of Mat and its nanoparticle formulations in addressing ALI, emphasizing the remarkable promise of natural compounds in ALI treatment. Through the application of network pharmacology, we have delineated the multitarget characteristics of Mat, underscoring its ability to influence critical signaling pathways, such as NF-κB and MAPK, which are integral to the inflammatory response. This approach not only confirms Mat’s multifaceted drug profile but also highlights the importance of merging traditional knowledge with modern scientific verification.

Our *in vivo* experiments further validate the effectiveness of Mat and its nanoparticle formulations, particularly the biomimetic PM@Mat-NPs, in mitigating the effects of ALI. The observed substantial decreases in inflammatory markers, vascular permeability, and pulmonary edema, coupled with the preservation of alveolar structure, collectively demonstrate the anti-inflammatory and protective properties of these formulations. These results underscore the potential of biomimetic nanoparticles as targeted delivery systems for anti-inflammatory agents, indicating broader applicability in the treatment of various inflammatory conditions. Besides, our work focuses on a pragmatic advancement: leveraging the mature platelet membrane biomimetic nanoparticle platform to deliver matrine—a well-characterized anti-inflammatory agent—specifically for ALI treatment. This “established carrier + known drug” strategy is designed to address the clinical gap of matrine’s poor targeting and potential systemic toxicity, rather than pursuing biomimetic carrier technology itself. As shown in our data, the platelet membrane coating endows matrine with enhanced lung accumulation (Figures 7B and 7C) and reduced off-target distribution, which significantly amplifies its inherent anti-inflammatory efficacy (e.g., inhibiting calcium influx, restoring mitochondrial function, and suppressing NLRP3 inflammasome; Figures 6, 7, and 8) while minimizing systemic exposure. This application-specific optimization—tailoring a mature biomimetic system to improve the therapeutic utility of a known drug in ALI—represents a practical innovation in translational research.

### Limitations of the study

(1) Animal Model Limitations: The study used only an LPS-induced ALI mouse model, which primarily mimics infectious inflammation. This model cannot fully represent the complex mechanisms of clinical ALI/ARDS, such as those caused by trauma, ischemia-reperfusion, or viral infection.
(2) Mechanistic Exploration Gaps: Although network pharmacology suggested matrine’s involvement in calcium and cAMP signaling pathways, and *in vitro* experiments confirmed its effects on calcium influx and mitochondrial function, specific molecular targets of PM@Mat-NPs (e.g., key proteins or receptors) were not validated.

Pharmacokinetic and Long-Term Safety Deficiencies: The study assessed only short-term (24-h) biodistribution and acute toxicity. It lacked systematic pharmacokinetic data (e.g., half-life, clearance rate) and long-term safety evaluations (e.g., chronic toxicity, immunogenicity, or organ damage after repeated administration), which are crucial for clinical translation.

## Resource availability

### Lead contact

Requests for further information should be directed to and will be fulfilled by the lead contact, Hua Jin (jinhua0413@gdmu.edu.cn).

### Materials availability

This study did not generate unique reagents. All materials used in this study will be made freely available upon request and the completion of applicable material transfer agreements. The information about reagents or other materials is provided in the key resources table.

### Data and code availability

• Data: Data reported in this article will be shared by the lead contact upon request.
• Code: This article does not report original code.
• Additional information: Any additional information required to reanalyze the data reported in this article is available from the lead contact upon request.

## Acknowledgments

This work was supported by the 10.13039/501100003453Natural Science Foundation of Guangdong Province (2022A1515140154), the Science Foundation of Dongguan Science and Technology Bureau (20231800940172), and the Guangdong Provincial Medical Research Fund (A2024676).

## Author contributions

Conceptualization: H.J.; methodology: Y.F., H.Z., X.C., J.L., Y.Y., and X.S.; writing – original draft: H.J. and Y.Z.; writing – review and editing: H.J. and Y.Z.; supervision: N.W., M.Y., L.L., Y.Z., and H.J.; project administration: Y.Z., Y.Y., J.L., R.L., N.W., and X.C.; funding acquisition: Y.F. and H.J.; all authors have read and agreed to the published version of the article.

## Declaration of interests

There are no conflicts to declare.

## STAR★Methods

### Key resources table

**Table:** 

REAGENT or RESOURCE	SOURCE	IDENTIFIER
**Antibodies**		

Matrine	Aladdin	https://www.rjmart.cn/
Poly(lactic-co-glycolic acid)	Sigma-Aldrich	https://www.rjmart.cn/
Dichloromethane	Sigma-Aldrich	https://www.rjmart.cn/
Dulbecco’s Modified Eagle Medium	Gibco BRL	Grand Island, NY, USA
Fetal bovine serum	Gibco BRL	Grand Island, NY, USA
Trypsin	Gibco BRL	Grand Island, NY, USA
Penicillin-streptomycin	Gibco BRL	Grand Island, NY, USA
2′,7′-dichlorofluorescein diacetate (DCFH-DA)	Sigma-Aldrich	St. Louis, MO, USA
Alanine transaminase and Aspartate transaminase	Nanjing Jiancheng Bioengineering Institute	https://www.rjmart.cn/
Lipopolysaccharides	Solarbio	https://www.rjmart.cn/

**Experimental models: Cell lines**		

Cell Counting Kit-8	Zeta Life	San Diego, CA, USA

**Experimental models: Organisms/strains**		

Female C57BL/6J mice	SPF Biotechnology Co., Ltd	https://www.rjmart.cn/

**Software and algorithms**		

Cytoscape	P. Shannon	https://cytoscape.org/
String	D. Szklarczyk	https://cn.string-db.org/
DAVID	W. Huang da	https://davidbioinformatics.nih.gov/

### Method details

#### Preparation of Mat-NPs

Mat-loaded nanoparticles (Mat-NPs) were prepared using an emulsification and solvent evaporation method, with modifications based on our previously published protocol.^17^ Briefly, 50 mg of Mat and 200 mg of poly(lactic-co-glycolic acid) (PLGA) were dissolved in 5 mL of dichloromethane to form the oil phase. This mixture was homogenized for 30 s on ice to ensure uniform dispersion. Subsequently, an aqueous phase containing 1% polyvinyl alcohol (PVA) was added to the oil phase, and the resulting mixture was homogenized for 3 min on ice to form a stable emulsion. The emulsion was then subjected to solvent evaporation at room temperature for 24 h to remove dichloromethane. The resulting nanoparticles were isolated by centrifugation at 12,000 rpm for 20 min at 4°C. The nanoparticles were washed three times with ultrapure water to remove any residual impurities. Finally, the harvested nanoparticles were lyophilized for 48 h to obtain a stable powdered form, which is suitable for storage and further experimentation.

#### Isolation of platelet membrane vesicles

Platelet membrane vesicles were isolated from anticoagulated whole blood. Briefly, 500 μL of EDTA-anticoagulated whole blood was centrifuged at 200 g for 10 min to obtain platelet-rich plasma (PRP). The PRP was then centrifuged at 1,800 g for 20 min at 4°C to pellet the platelets. The supernatant was discarded, and the platelets were washed twice with phosphate-buffered saline (PBS) to remove any residual plasma proteins. The platelets were then suspended in deionized water containing a protease inhibitor cocktail to prevent protein degradation. The suspension was subjected to three cycles of freeze-thawing (−80°C for 1 h, followed by thawing at room temperature) to disrupt the platelet membranes. After the freeze-thaw cycles, the sample was sonicated for 30 s to further disintegrate the membranes and release the platelet membrane vesicles.

#### Preparation of PM@Mat-NPs

To fabricate platelet membrane-coated Mat nanoparticles (PM@Mat-NPs), the isolated platelet membrane vesicles were added to the pre-synthesized Mat-NPs. The mixture was extruded through a hand extruder (Simon Machinery Co., China). equipped with a polycarbonate membrane (pore size 200 nm) at least three times. This extrusion process ensured the uniform coating of Mat-NPs with platelet membranes, resulting in the formation of well-defined PM@Mat-NPs. The PM@Mat-NPs were then collected and used for subsequent characterization and biological assays.

#### Characterization of PM@Mat-NPs

The size distribution and zeta potential of the nanoparticles were measured using a nano particle analyzer (SZ-100, Horiba Scientific, USA). These parameters are critical for understanding the physical properties of the nanoparticles, as size influences their circulation time in the body and their ability to penetrate tissues, while zeta potential affects their stability in solution. The morphology of the nanoparticles was examined using scanning electron microscopy (SEM, Philips Co., Holland). SEM imaging provided high-resolution images, allowing for direct observation of the shape and surface characteristics of the nanoparticles. Also, the transmission electron microscopy (TEM) images reveal that the cell membrane - coated matrine nanoparticles are surrounded by a uniform layer. This phenomenon arises from the difference in density between the cell membrane and the nanoparticles. In contrast, the uncoated matrine nanoparticles lack such a uniform matrix layer.

The encapsulation efficiency of Mat within the nanoparticles was assessed using an ultraviolet-visible spectrophotometer (UV 6000). Mat, Mat-NPs, and PM@Mat-NPs were diluted 10-fold to a concentration of 1 mg/mL. The loading efficiency and concentration of Mat in the nanoparticles were measured using a microplate reader (Infinite 200 Pro, Switzerland) at 220 nm. The concentration of Mat was determined based on a pre-established standard curve, enabling accurate quantification of the drug loading within the nanoparticles.

The physicochemical properties of the PM@Mat-NPs were further investigated using Fourier transform infrared (FT-IR) spectroscopy (Thermo Nicolet 6700, USA). FT-IR spectroscopy provided information on the chemical bonds and functional groups present in the nanoparticles, confirming the successful encapsulation of Mat and the integrity of the nanoparticle structure.

The retention of membrane proteins from the platelet membranes was analyzed using sodium dodecyl sulfate-polyacrylamide gel electrophoresis (SDS-PAGE). Protein samples were mixed with loading buffer (Beyotime, China) and denatured at 95°C to unfold the proteins and expose their polypeptide chains. A total of 30 μg of protein samples, including those from isolated platelet membranes (PM) and PM@Mat-NPs, were separated on a 10% SDS-PAGE gel. After electrophoresis, the gel was stained with Coomassie Blue for 2 h to visualize the protein bands, followed by overnight decolorization to remove excess stain. This analysis provided insights into the protein composition of the platelet membranes and their presence on the surface of the PM@Mat-NPs, which is essential for understanding the biological properties of the biomimetic nanoparticles.

#### Determination of intracellular calcium ions in endothelial cells (ECs)

The dynamic changes in intracellular calcium ion concentrations within endothelial cells were assessed using a calcium ion assay kit. This assay was designed to evaluate the effects of Mat or Mat-loaded nanoparticles (Mat-NPs) on lipopolysaccharide (LPS)-induced calcium ion fluctuations. Endothelial cells were pre-treated with Mat or Mat-NPs for 12 h, followed by exposure to LPS for an additional 12 h. The assay relies on calcium-sensitive fluorescent probes, such as Fluo-4 a.m., which exhibit minimal fluorescence in their native state. Upon internalization by cells, these probes are hydrolyzed by intracellular esterases to produce Fluo-4, which has a high affinity for calcium ions and emits green fluorescence upon binding. The fluorescence intensity is directly proportional to the intracellular calcium ion concentration. Mean fluorescence intensity (MFI) was quantified across different treatment groups to analyze the impact of Mat and Mat-NPs on LPS-induced calcium ion changes in ECs.

#### F-actin staining in endothelial cells (ECs)

Endothelial cells were seeded onto cell slides and cultured until they reached 50–70% confluence. Cells were pre-treated with Mat or Mat-NPs for 12 h, followed by exposure to LPS for another 12 h. The culture medium was then aspirated, and cells were washed twice with pre-warmed 1×PBS (pH 7.4) to remove residual medium and contaminants. Cells were fixed with 3% formaldehyde at room temperature for 30 min to preserve structural integrity. After fixation, cells were washed 2–3 times with PBS (10 min per wash). Cells were permeabilized with 0.1% Triton X-100 in PBS for 5 min to allow entry of fluorescently labeled phalloidin. Following permeabilization, cells were washed three times with PBS (10 min per wash). Fluorescently labeled phalloidin working solution (e.g., 100 μL/well in a 12-well plate) was added, and cells were stained in the dark for 90 min. Cells were then washed three times with PBS (5 min per wash) to remove excess phalloidin. For nuclear staining, DAPI solution (e.g., 100 μL/well) was added and incubated at room temperature for 5 min. After nuclear staining, cells were washed twice with PBS (5 min per wash). Excess liquid was removed, and a fluorescent mounting medium was added. A coverslip was placed over the cells, and they were observed under a fluorescence or confocal microscope with appropriate filters.

#### Mitochondrial membrane potential assay in endothelial cells (ECs)

Endothelial cells were seeded onto culture dishes or slides and grown to 70–80% confluence. Cells were pre-treated with Mat or Mat-NPs for 12 h, followed by exposure to LPS for another 12 h. The culture medium was aspirated, and JC-1 staining solution (prepared according to the manufacturer’s instructions) was added to cover the cells. Cells were incubated with JC-1 at 37°C for 15–30 min in the dark. JC-1 is a cationic dye that accumulates in mitochondria in a potential-dependent manner. In healthy mitochondria with high membrane potential, JC-1 forms aggregates that emit red fluorescence (excitation/emission filters: 585/590 nm), while in mitochondria with low membrane potential, JC-1 exists as monomers and emits green fluorescence (excitation/emission filters: 488/530 nm). After incubation, the staining solution was removed, and cells were washed twice with PBS to remove unbound JC-1. Cells were then observed under a fluorescence microscope using the appropriate excitation/emission filters.

#### Apoptosis assay in endothelial cells (ECs) using TUNEL staining

Endothelial cells were seeded onto culture dishes or slides and grown to 70–80% confluence. Cells were washed with PBS to remove residual culture medium. The TUNEL reaction mixture (prepared according to the kit instructions) was added to cover the cells, and they were incubated at 37°C for 60 min in the dark. After incubation, the reaction mixture was removed, and cells were washed twice with PBS. For fluorescence microscopy, PBS was added to the cells, and they were observed under a fluorescence microscope using excitation/emission filters of 488/530 nm for green fluorescence (TUNEL-positive cells). Optionally, nuclei were counterstained with DAPI (excitation/emission filters: 358/461 nm) to visualize the total cell number. This assay allowed for the quantification of apoptotic cells in endothelial cell cultures, providing insights into the anti-apoptotic effects of Mat or Mat-NPs.

#### Data acquisition for ALI gene expression profiles

To obtain gene expression profiles and matrices related to ALI, the terms “*mucosal acute lung injury*” or “*inflammatory acute lung injury*” were searched in the GeneCards、OMIM、DisGeNet database. Key dataset characteristics (e.g., sample size, experimental grouping, tissue type) were systematically recorded. Gene probe information was extracted from the data download column, and all gene IDs in the matrix file were mapped to corresponding probes through annotation matching.^16^

#### Target identification for Mat

Database Mining: Target genes of Mat were initially retrieved from the Traditional Chinese Medicine Systems Pharmacology (TCMSP) database. Cross-Validation: Using the UniProt KB function, related genes were further validated in the UniProt database to ensure accuracy. In Silico Prediction: Potential Mat targets were additionally predicted using the Swiss Target Prediction tool. Integration: All targets from the two sources were combined and deduplicated to establish a comprehensive Mat target gene set.

#### Differential gene analysis via GEO2R

GEO2R, a web-based analytical tool, was employed to identify differentially expressed genes (DEGs) across multiple experimental conditions in ALI datasets. The criteria for DEGs were set as |logFC| > 1.0 and *p* < 0.05. Following data filtering via GEO2R computations, volcano plots and dimensionality reduction graphs (e.g., principal component analysis, PCA) were generated to visualize significant expression changes.

#### Functional enrichment analysis

The DAVID bioinformatics resource was used to perform Kyoto Encyclopedia of Genes and Genomes (KEGG) pathway enrichment analysis on both DEGs from ALI datasets and Mat target genes. This analysis aimed to uncover shared biological processes and molecular pathways underlying ALI pathogenesis and Mat’s potential mechanisms of action, with references to prior literature.

### Experimental model and study participant details

#### Animals

Female C57BL/6J mice, aged 8 weeks, were obtained from SPF Biotechnology Co., Ltd. (Beijing, China). All the animals were fed in the SPF animal center of Guangdong Medical University. All experimental procedures were conducted in strict compliance with the guidelines for the use of laboratory animals established by the National Institutes of Health. The study was approved by the Institutional Animal Care and Use Committee of Guangdong Medical University and adhered to the ethical guidelines and regulations set forth by the Animal Ethics Committee of Guangdong Province, China. Experimental procedures using mice in this study were reviewed and approved by the ethical review board of Guangdong Medical University, and all the experiments were performed in accordance with relevant guidelines and regulations of Animal Ethics Committee of Guangdong Province, China. Animals and protocol were approved by the Ethics Committee of Guangdong Medical University (GDY2002004). In this study, only female mice were employed, and male mice were not requisitioned.

#### Cell lines

Human endothelial cells were obtained from Fuheng Biology (Shanghai, China). These cells were cultured in DMEM (Gibco, USA) supplemented with 10% fetal bovine serum (FBS, Gibco), 100 μg/mL streptomycin, and 100 IU/mL penicillin. The cultures were maintained in a humidified incubator with 5% CO_2_ at 37°C.

The cell lines used in this work have not been authenticated and have not undergone mycoplasma contamination testing, and the absence of contamination was initially determined based on the researchers' experience.

#### ALI mouse model and experimental groups

ALI was induced in 8-week-old female C57BL/6J mice via intraperitoneal injection of lipopolysaccharide (LPS, 3.5 mg/kg body weight). Three hours post-LPS administration, mice were randomly assigned to distinct experimental cohorts. The control group received an intranasal injection of saline, while the experimental groups were intranasally with one of three formulations of Mat (free Mat, Mat-NPs and PM@Mat-NPs) at a dose of 10 mg/kg body weight. After 24 h, mice were euthanized, and samples, including blood, bronchoalveolar lavage fluid (BALF), and lung tissues, were collected for further analysis.

#### *In vivo* targeting evaluation

To assess the *in vivo* targeting efficacy of platelet membrane (PM)-coated Mat nanoparticles in an ALI mouse model, the IVIS (*In Vivo* Imaging System) was employed. ALI was induced using an appropriate method, such as intratracheal instillation of LPS. Mice were anesthetized with isoflurane to ensure comfort during imaging. Free Mat or PM-coated Mat nanoparticles were labeled with a near-infrared (NIR) fluorescent dye (e.g., indocyanine green (ICG)), and administered intranasally. Mice were then placed in the IVIS chamber, and imaging was performed immediately after injection to establish a baseline. Subsequent imaging was conducted at 6, 12, and 24 h to track nanoparticle accumulation and distribution in the lungs. Fluorescence intensity in regions of interest (ROIs) within the lungs was quantified using IVIS software, allowing for the evaluation of targeting efficiency and nanoparticle retention in injured lung tissue.

#### Pro-inflammatory cytokine assay

The levels of pro-inflammatory cytokines, including IL-1β and TNF-α, in plasma and bronchoalveolar lavage fluid (BALF) were quantified using enzyme-linked immunosorbent assay (ELISA) kits (Jiangsu Meimian Industrial Co., Ltd.), following the manufacturer’s protocols.

#### Histological analysis

Lungs collected from mice were initially fixed by instilling 10% formalin through tracheal catheterization at a *trans*-pulmonary pressure of 15 cm H_2_O for 5 min to preserve tissue structure. The lungs were then further fixed in 10% formalin at room temperature for 48 h, followed by embedding in paraffin. Paraffin-embedded lungs were sectioned into 5 μm-thick slices, which were subsequently stained with hematoxylin and eosin (H&E), NLRP3 antibody, and TUNEL antibody for histological evaluation.

#### Biosafety assessment

Histopathological sections of major organs, including the heart, liver, spleen, and kidneys, were prepared and stained with hematoxylin and eosin (H&E). Additionally, the levels of ALT and AST in liver homogenates were measured using commercially available assay kits (C010-2-1, Nanjing Jiancheng Bioengineering Institute, China) to evaluate potential organ toxicity.

### Quantification and statistical analysis

Data are presented as the mean ± standard error of the mean (SEM). Statistical significance was assessed using one-way analysis of variance (ANOVA) followed by Games-Howell post hoc analysis for multiple-group comparisons. For comparisons between two groups, the two-tailed unpaired Student’s *t* test was employed.
